# Pharmacokinetics and bioavailability of a new long-acting insulin analog in healthy Chinese volunteers: an open, randomized, single-dose, two-period and two-sequence cross-over study

**DOI:** 10.3389/fphar.2023.1294810

**Published:** 2023-12-22

**Authors:** Ke-Guang Chen, Ye-Hui Zhang, Pan-Pan Ye, Xue-Hu Gao, Lin-Lin Song, Hai-Yan Zhou, Qian Li, Fu-Rong Zhao, Jin-Yi Shi, Xin-Mei Yang, Kai Shen, Sheng Feng, Wei Zhao

**Affiliations:** ^1^ Department of Clinical Pharmacy, The First Affiliated Hospital of Shandong First Medical University & Shandong Provincial Qianfoshan Hospital, Shandong Engineering and Technology Research Center for Pediatric Drug Development, Shandong Medicine and Health Key Laboratory of Clinical Pharmacy, Jinan, China; ^2^ Jiangsu Hengrui Medicine Co., Ltd., Lianyungang, Jiangsu, China; ^3^ Department of Clinical Pharmacy, Key Laboratory of Chemical Biology Ministry of Education, School of Pharmaceutical Sciences, Cheeloo College of Medicine, Shandong University, Jinan, China

**Keywords:** INS068 injection, pharmacokinetics, relative bioavailability, healthy subjects, insulin analog

## Abstract

**Objectives:** INS068 is a novel, soluble, and long-acting insulin analog. In this study, we evaluated the pharmacokinetics and relative bioavailability of two formulations of INS068 in healthy Chinese subjects: a reference formulation packaged in vials and administered via syringe (R), and a test formulation packaged and administered via pen injector (T).

**Methods:** A randomized, open-label, two-period, two-sequence crossover study was conducted with 24 healthy Chinese subjects. Subjects were randomized and administered subcutaneously in the abdomen at 0.4 U/kg of test or reference INS068 injection according to an open crossover design. INS068 concentrations in the serum were measured using LC-MS/MS, and the pharmacokinetic parameters of maximum concentration (C_max_) and area under the concentration-time curve (AUC_0–t_ and AUC_0–∞_) were used to evaluate relative bioavailability.

**Results:** After a single dose at 0.4 U/kg, the median T_max_ of INS068 was 12 h for both formulations, and the mean t_1/2_ for T and R was 13.0 h and 12.6 h, respectively. The geometric means of C_max_ and AUC_0–∞_ were 3.99 nmol/L and 120 h·nmol/L for the T, and 4.05 nmol/L and 117 h·nmol/L for the R, respectively. The geometric mean ratios of C_max_, AUC_0–t_ and AUC_0–∞_ of T over R were 98.7% (90% CI: 92.7%–105.2%), 102.6% (90% CI: 100.0%–105.3%) and 102.8% (90% CI: 100.1%–105.5%).

**Conclusion:** The overall PK profile of the two formulations of INS068 injection was comparable in healthy subjects, and the pen injector of INS068 had adequate safety and tolerability, supporting it as a new formulation in a phase III study and bridging PK data from early phase clinical trials.

**Clinical Trial Registration:** clinicaltrials.gov, identifier: NCT05336071

## 1 Introduction

Diabetes mellitus (DM) is a chronic metabolic disorder characterized by hyperglycemia due to insulin deficiency, insulin resistance, or both. Long-term hyperglycemia is associated with the damage, dysfunction, and failure of various organs, especially the kidneys, eyes, nerves, heart, and blood vessels (American Diabetes Association, 2013). DM is generally classified according to etiological factors, with type 1 diabetes mellitus (T1DM) and type 2 diabetes mellitus (T2DM) constituting the majority of cases (UK Prospective Diabetes Study Group, 1998; Holman et al., 2008; Hemmingsen et al., 2013). According to the International Diabetes Federation, there were 382 million people with diabetes in 2013 globally, and the number is expected to rise to 592 million by 2035 (Guariguata et al., 2014). The lifetime risk of diabetes for an average 20-year-old American person in the United States (US) increased from 20.4% for men and 26.7% for women from 1985-1989 to 40.2% for men and 39.3% for women based on 2011 data (Mobasseri et al., 2020). In other words, two out of every five Americans entering adulthood can expect to develop diabetes during their lifetime (Gregg et al., 2014; Lipscombe, 2014).

INS068 is a novel, soluble, long-acting insulin analog developed by Jiangsu Hengrui Pharmaceuticals Co., Ltd., intended to cover basal insulin requirements in patients with T1DM and T2DM. INS068 was designed based on desB30 human insulin, to exert stable and long-acting blood glucose-lowering effects. Nonclinical and clinical trials have demonstrated that INS068 injection has favorable safety profiles and exhibits pharmacodynamics comparable to those of IDeg (US Food and Drug Administration, 2015).

During the clinical development process, the drug substance (DS) and drug product (DP) of INS068 underwent significant changes. INS068 injection, used in Phase 1 and Phase 2 studies so far, was produced with manufacturing process A, packaged in a glass vial, and administered with an insulin syringe, whereas INS068 was planned for use in upcoming Phase 3 clinical trials and for future commercialization is produced with process B and packaged in and administered via a pen injector. Therefore, evaluation of any potential impact of manufacturing process and package changes on the bioavailability and overall PK properties of INS068 is warranted. This study aimed to evaluate the pharmacokinetics and relative bioavailability of INS068 injection between the two formulations in healthy subjects.

## 2 Subjects and methods

### 2.1 Subjects

This study was conducted from 12 May 2022, to 30 May 2022, at the Clinical Research Center, Shandong Provincial Qianfoshan Hospital, China. Twenty-four healthy male volunteers were recruited based on inclusion and exclusion criteria. Healthy volunteers aged between 18 and 55 years with a body mass index (BMI) between 18 and 27 kg/m^2^ were eligible to participate. This study was approved by the National Drug Clinical Trial Institution and Research Ethics Committee (REC) of Shandong Provincial Qianfoshan Hospital and Human Genetic Resource Administration of China (HGRAC), and was carried out in accordance with the International Conference on Harmonization (ICH) Good Clinical Practice guidelines (ICH, 2016) and the Declaration of Helsinki (World Medical Association, 2013). Written informed consent was obtained from all the participants prior to the initiation of any study-related activities.

### 2.2 Study design

This was a randomized, open-label, two-period, two-sequence crossover study designed to evaluate the PK of two different INS068 injection formulations in healthy subjects. INS068 used in Phase 1 and Phase 2 studies, manufactured with process A, packaged in vial, and administered with a syringe, was designated as the reference (R) product, and INS068 used in Phase 3 studies, manufactured with process B, packaged and administered with pen injector, was designated as the test (T) product.

In the first phase, prior to the commencement of dosing, screening and physical examination were performed within 14 days. The screening procedures included demographic and medical history data collection, general physical examination, and electrocardiography. Laboratory investigations, such as hematology, biochemistry, and urine analysis, were also performed.

**FIGURE 1:**
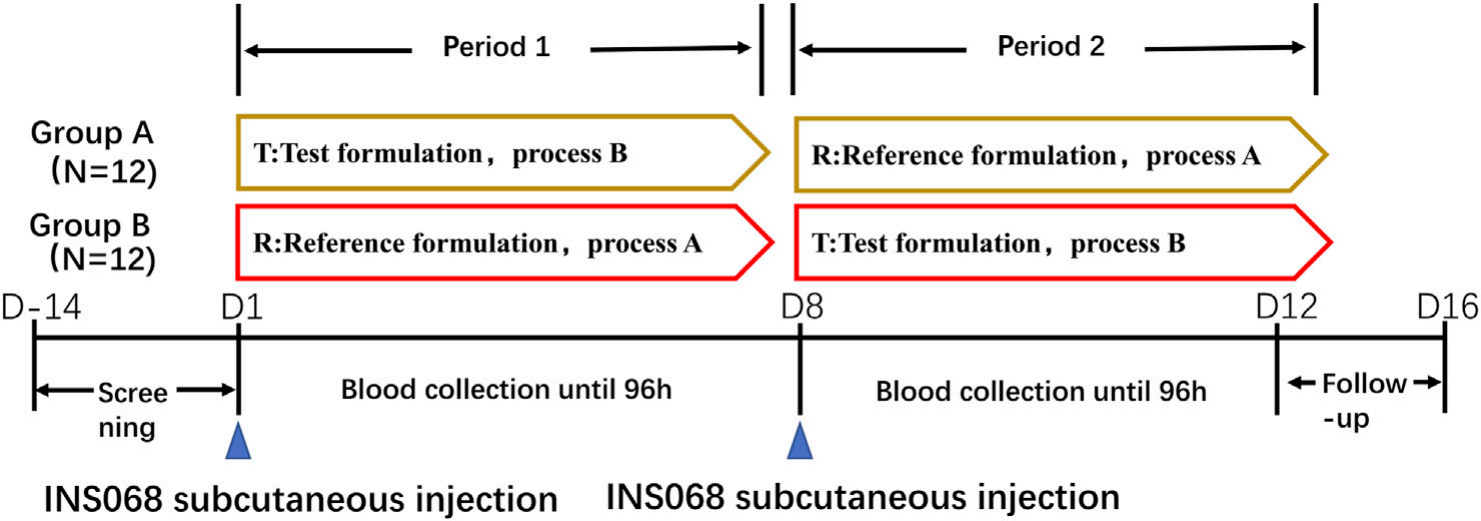
Flow diagram showing the trial design.

The admitted subjects were enrolled and randomized in a 1:1 ratio into two study groups (Figure 1). They were fed a standard light diet in the evening and fasted overnight after dinner for over 10 h. On the morning of study Day 1, the subjects underwent venipuncture (indwelling needles) for infusion of 10% glucose solution and PK sample collection. According to randomization, the subjects received either the reference or test formulation of the INS068 injection at approximately 8 a.m., administered subcutaneously in the abdomen at 0.4 U/kg. A standard breakfast meal was provided approximately 1 h before INS068 administration and water was allowed if needed. Blood samples were collected according to the protocol detailed below to measure INS068 concentrations in the serum. On the morning of study Day 8, the subjects underwent the same procedures as those on Day 1 except that they received a different formulation of INS068 than on Day 1.

### 2.3 Pharmacokinetics

Blood samples were collected 1 h before INS068 administration and at 2, 4, 6, 8, 10, 12, 14, 16, 18, 24, 48, 72, and 96 h after administration (14 blood samples in total) to determine the concentration of INS068 in the serum. Immunogenicity samples were collected within 1 h before INS068 administration and then at 16 and 96 h after administration for the analysis of anti-INS068 antibodies on Day 1 and Day 8. Blood samples were centrifuged after standing at room temperature for 30 min to 1 h, and the harvested serum was frozen and maintained at −60°C to −90°C until further analysis. The serum sample was stable for 24 h at room temperature, stable for 144 h at 2°C–8°C, and stable for 748 days at −70°C. Serum INS068 concentrations and anti-INS068 antibodies were measured by Labcorp Pharmaceutical Research & Development (Shanghai) Co., Ltd. (Shanghai, China). Serum INS068 concentrations were measured by LC-MS/MS. The analysis system consists of the HPLC-30AD system (Shimadzu, Japan) and the Sciex Triple Quad 6500 + triple quadrupole tandem mass spectrometry (AB Science, Foster City, California). Appropriate amounts of serum samples, internal standards, and buffer were mixed and transferred to a solid phase extraction (SPE) plate. Water and organic solvent were added as eluent. The collected eluent was blown dry by nitrogen gas, and a complex solution of organic solvent was added. The mixture was taken for injection. The retention time of INS068 was 0.93 min. The detection method has been validated, with accuracy and precision of less than 10%. The lower limit of quantification was 0.04 nmol/L, and the concentration range of the standard curve was 0.04–8 nmol/L.

### 2.4 Assessments

Assessments were based on the PK parameters of INS068 injected by subcutaneous injection of the two formulations in healthy subjects: AUC_0-t_, C_max_, AUC_0-∞_, time to maximum observed concentration (T_max_), terminal elimination half-life (t_1/2_), apparent volume of distribution (V/F), and apparent clearance (CL/F). The safety assessments included adverse events (AEs), vital signs, physical examination, laboratory tests, 12-lead electrocardiography (ECG), anti-INS068 antibodies, injection site reactions, and hypoglycemic events.

### 2.5 Statistical methods

Statistical analyses were performed based on the non-compartmental method using Phoenix WinNonlin (version 8.1) to identify the PK parameters C_max_, AUC_0-t_, AUC_0-∞_ (if applicable), t_1/2_, T_max_, CL/F, and Vz/F of the INS068 injection. SAS software (version 9.4; SAS Institute Inc., Cary, North, USA) was used for statistical analyses. The point estimate with a 90% confidence interval of the least squares mean difference (T over R) was calculated for each PK parameter of C_max_, AUC_0-t_, and AUC_0-∞_ after natural logarithmic transformation based on the PK parameter set. The antilogarithmic transformation was also performed to calculate the point estimates of the geometric mean ratios (T over R) and the corresponding 90% confidence intervals.

## 3 Results

### 3.1 Demographic characteristics of the subjects

A total of 56 subjects were screened, of which 24 were enrolled in the study and 32 failed screening. All 24 enrolled subjects completed the trial, including dosing, PK blood collection, safety observation, and follow-up. All subjects were Chinese. Their mean age was 30.3 years (range, 19–48 years), mean height (± standard deviation) was 172.52 ± 5.428 cm, mean weight (± standard deviation) was 68.17 ± 7.432 kg, and mean BMI (± standard deviation) was 22.863 ± 1.848 kg/m^2^ (Table 1).

**TABLE 1:** Summary of demographic information.

		Total (N = 24)
**Age**(**year**)	N	24
	Mean (SD)	30.3 (7.26)
	Median	29.5
	Min, Max	19, 48
**Sex, n (%)**	Male	24 (100.0)
	Female	0
**Race, n (%)**	Han nationality	24 (100.0)
	Others	0
**Height (cm)**	N	24
	Mean (SD)	172.52 (5.428)
	Median	171.00
	Min, Max	163.5, 181.5
**Weight (kg)**	N	24
	Mean (SD)	68.17 (7.432)
	Median	67.40
	Min, Max	57.4, 83.6
**BMI (kg/m** ^**2**^ **)**	N	24
	Mean (SD)	22.863 (1.8483)
	Median	23.100
	Min, Max	19.60, 26.40

Decimal places of data are determined based on the decimal places of the original value: the Mean and Median values are accurate to one decimal place more than the original value; the SD, values are accurate to two decimal places more than the original value; the MIN, and MAX are accurate to the same number of decimal places as the original value. The maximum number of decimal places is 4.

### 3.2 Pharmacokinetics

**FIGURE 2:**
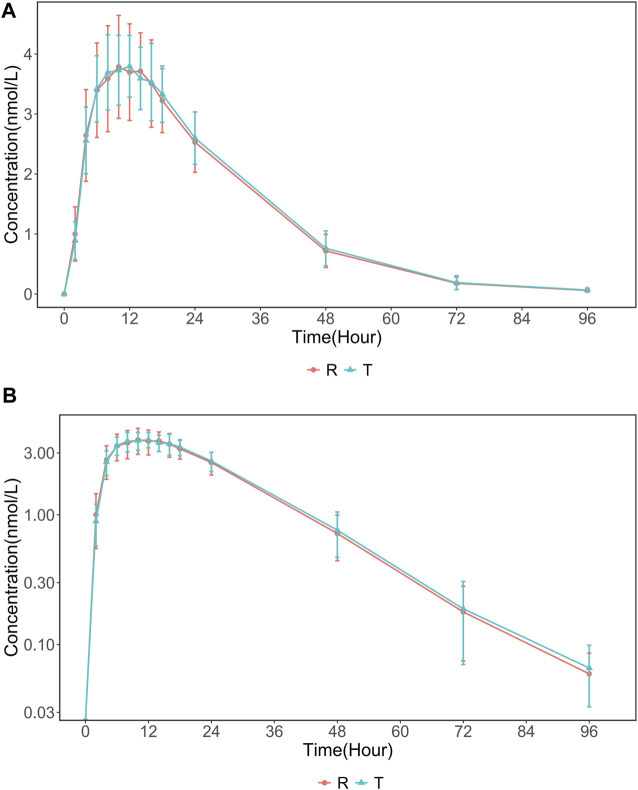
The mean (SD) serum INS068 Concentration-Time Profiles after Single Subcutaneous two formulations INS068 in Healthy Male Subjects: **(A)** non-log transformed data (mean ± SD) and **(B)** semi-log transformed data.

The PK population included 24 subjects who were enrolled in the trial. Serum concentrations in the 24 subjects were quantified and analyzed. The mean 96 h serum INS068 concentration-time profiles for the two formulations are shown in Figure 2. The descriptive statistics of the PK parameters of the reference (R) and test (T) formulations of INS068 are shown in Table 2. Following administration of a single dose of 0.4 U/kg, the PK properties of the two INS068 formulations were similar. The median T_max_ was 12 h for both T and R, and the mean t_1/2_ for T and R was 13.0 h and 12.6 h, respectively. The geometric means (GeoMean) of C_max_ and AUC_0-∞_ were 3.99 nmol/L and 120 h·nmol/L for the T, 4.05 nmol/L and 117 h·nmol/L for the R, respectively. The GeoMean ratios (T over R) of C_max_, AUC_0-t_ and AUC_0-∞_ were 98.7% (90% CI: 92.7%–105.2%), 102.6% (90% CI: 100.0%–105.3%) and 102.8% (90% CI: 100.1%–105.5%), respectively (Table 3).

**TABLE 2:** Summary of the pharmacokinetic parameters of two formulations of INS068 in healthy male subjects.

Parameter (unit)	Statistical variable	R (vial, n = 24)	T (pen, n = 24)
**t**_**1/2**_ **(h)**	Mean (SD)	12.6 (1.73)	13.0 (2.23)
	CV%	13.7	17.1
	GeoMean	12.5	12.9
**T**_**max**_ **(h)**	Median	12.0	12.0
	Min	6.00	6.00
	Max	16.0	18.0
**C**_**max**_ **(nmol/L)**	Mean (SD)	4.13 (0.869)	4.03 (0.563)
	CV%	21.0	13.9
	GeoMean	4.05	3.99
**AUC**_**0-t**_ **(h·nmol/L)**	Mean (SD)	117 (17.3)	120 (12.7)
	CV%	14.8	10.6
	GeoMean	116	119
**AUC**_**0-∞**_ **(h·nmol/L)**	Mean (SD)	118 (17.4)	121 (12.9)
	CV%	14.7	10.7
	GeoMean	117	120

Abbreviations: T = test formulation, process B; R = reference formulation, process A. t_1/2_, terminal elimination half-life; T_max_, time to Cmax; C_max_, maximum serum concentration; AUC_0-t_, area under the serum concentration-time curve from time zero to last measurable concentration; AUC_0-∞_, area under the serum concentration-time curve from time zero to infinity. GeoMean, Geometric Mean.

The pharmacokinetic data results are retained with three significant figures.

**TABLE 3:** Summary of the statistical comparisons of the pharmacokinetic parameters of two formulations of INS068 injection.

Parameter (units)	Comparison	GeoMean ratio (%)	GeoMean ratio 90% CI
**C**_**max**_ **(nmol/L)**	T vs. R	98.74	92.70–105.17
**AUC**_**0-t**_ **(h·nmol/L)**	T vs. R	102.64	100.00–105.34
**AUC**_**0-∞**_ **(h·nmol/L)**	T vs. R	102.77	100.09–105.52

Abbreviations: T = test formulation, process B; R = reference formulation, process A. c_max_, maximum serum concentration; AUC_0-t_, area under the serum concentration-time curve from time zero to last measurable concentration; AUC_0-∞_, area under the serum concentration-time curve from time zero to infinity. GeoMean, Geometric Mean.

### 3.3 Safety and tolerability

Both the test and reference formulations of the INS068 injection were well tolerated. All the enrolled subjects completed the study according to the protocol. None of the subjects discontinued or withdrew from the study because of safety issues.

The overall incidence of treatment-emergent adverse events (TEAE) was 29.2% (7/24), of which 16.7% (4/24) were associated with the test formulation, including blood triglyceride levels increased, electrocardiogram ST-T change, and blood creatine phosphokinase increased; 12.5% (3/24) were associated with the reference formulation, including alanine aminotransferase increased, blood pressure increased, dizziness, and diarrhea. The summary of TEAEs is shown in Table 4. All TEAEs were mild in severity (asymptomatic or mild symptoms and no corrective treatment was required). There were no serious adverse events (SAE), deaths, or adverse events of special interest (AESI), nor were there any adverse events that led to dose adjustment or corrective treatment. All TEAEs recovered by the end of the trial, except for one event (increased blood triglycerides) with an unknown outcome.

**TABLE 4:** Summary of treatment-emergent adverse events (TEAEs) by system organ class and preferred terms.

System organ class preferred term	T (N = 24) n (%)	R (N = 24) n (%)	Total (N = 24) n (%)
Number of subjects who experienced at least one TEAE	4 (16.7)	3 (12.5)	7 (29.2)
Investigations	4 (16.7)	2 (8.3)	6 (25.0)
Blood triglycerides increased	3 (12.5)	0	3 (12.5)
Alanine aminotransferase increased	0	1 (4.2)	1 (4.2)
Electrocardiogram ST-T change	1 (4.2)	0	1 (4.2)
Blood pressure increased	0	1 (4.2)	1 (4.2)
Blood creatine phosphokinase increased	1 (4.2)	0	1 (4.2)
Nervous system disorders	0	1 (4.2)	1 (4.2)
Dizziness	0	1 (4.2)	1 (4.2)
Gastrointestinal disorders	0	1 (4.2)	1 (4.2)
Diarrhea	0	1 (4.2)	1 (4.2)

Abbreviations: T = test formulation, process B; R = reference formulation, process A. MedDRA (v25.0) was used for coding. n: Number of subjects who experienced at least one adverse event. %: Percentage of subjects who experienced at least one adverse event. SOCs, are sorted in the total column by the frequency of occurrence from high to low. Within a SOC, PTs, are sorted by the frequency of occurrence from high to low. The normal range of continuous variables of investigations is consistent with the normal range used in clinical practice.

After administration of the test and reference formulations of INS068, there were no significant changes in laboratory results on average, vital signs, ECGs, or physical examination. Nine subjects experienced one hypoglycemic episode after injection of INS068 (including T and R), with an incidence rate of 37.5% (9/24). All hypoglycemic episodes had a level 1 severity according to the 2022 ADA classification (Davies et al., 2022). To date, no injection site reactions have been reported. The Anti-INS068 antibody was detected in 1 of the 24 subjects (4.2%) prior to the administration of INS068. After dosing at 0.4 U/kg, no ADA was detected in the serum of any of the INS068-treated subjects.

## 4 Discussion

INS068 is a novel long-acting human insulin analog. This mode of action is identical to that of human insulin and other insulin analogs, as they all act through the same insulin receptor. INS068 was packaged in a glass vial and administered with an insulin syringe when it was used in Phase 1 and Phase 2 studies. Two Phase 1 studies were conducted in healthy subjects in the US and China respectively. INS068 has slow absorption and metabolism, with T_max_ of 12–18 h and t_1/2_ of 13.3–21.4 h. The AUC_0-∞_ was directly proportional to the dose, and the mean AUC_0-∞_ of healthy male subjects in China was 6%–21.7% higher than that in the US. A Phase 1 study conducted in the US type 1 diabetes subjects showed that the PK characteristics of INS068 were similar to IDeg in the dose range of 0.4–0.8 U/kg. An international multicenter Phase 2 study showed that INS068 has good safety, tolerability, and efficacy comparable to IDeg, with glycation levels reduced by 0.98% and 0.97%, respectively. However, INS068 was planned to be packaged and administered via pen injector for upcoming Phase 3 clinical trials and future commercialization. Therefore, the present clinical study compared the PK properties of two INS068 formulations in healthy subjects.

Overall, no statistically significant differences in PK characteristics were observed between the formulations in this study. According to the study results, the PK parameters of the R and T products of INS068 were comparable, and the GeoMean ratios (T over R) of C_max_, AUC_0-t_ and AUC_0-∞_ and their 90% confidence interval (CI) were between 80% and 125% (Table 3). The GeoMean ratios (T over R) and 90% CI of C_max_, AUC_0-t_ and AUC_0-∞_ within each group were also calculated as an additional assessment, and all of them fell into the range of 80%–125% (Group A: 92.16%–97.15% for GeoMean ratios and 84.36%–101.23% for 90% CI; Group B: 108.75%–109.24% for GeoMean ratios and 97.82%–121.98% for 90% CI). The CV (%) of the new formulation (T) was lower than that of the old formulation (R). Both the test and reference formulations of the INS068 injection were well tolerated. The results demonstrated that neither the changes in the processes of DS and DP nor the packaging and delivery device affected the bioavailability or overall PK properties of INS068 injection, thereby qualifying the use of the new INS068 product in the upcoming phase 3 studies and for commercialization purposes.

This study has some limitations. First, pharmacodynamics were not investigated in this study, lacking the glucose clamp design, which makes it difficult to reflect the effect of changes in blood glucose in the PK profile of INS068. On the other hand, this study was conducted in healthy male subjects, rather than patients with type 1 diabetes (Korsatko et al., 2013), to include a relatively homogenous cohort of subjects to facilitate the detection of differences between the two formulations, in accordance with regulatory standards (US Food and Drug Administration, 2009; Committee for Medicinal Products for Human Use, 2010). With the inclusion of healthy subjects, a multiple-dose study with a clinically relevant dose would not have been acceptable, due to the risk of hypoglycaemia. We randomized subjects to fixed-dose levels of 0.4 U/kg of INS068 and single dose, which clearly does not reflect the therapeutic use of INS068. In actual clinical practice, the dose of INS068 must be titrated and adjusted to meet individual insulin needs without compromising safety, especially during hypoglycemic events. This is because, in clinical studies, hypoglycemic episodes caused by a lack of personalized basic insulin doses may be considered harmful to subjects.

## 5 Conclusion

The PK profiles of the two INS068 formulations were comparable following a single administration to healthy male subjects. Both the test and reference formulations of the INS068 injection were safe and well tolerated. The results support the use of the pen injector in a phase III study and bridge PK data from early phase clinical trials.

## Data Availability

The raw data supporting the conclusion of this article will be made available by the authors, without undue reservation.

## References

[B1] American Diabetes Association (2013). Diagnosis and classification of diabetes mellitus. Diabetes care 36 (1), S67–S74. 10.2337/dc13-S067 23264425 PMC3537273

[B2] Committee for Medicinal Products for Human Use (2010). Guideline on the investigation of bioequivalence. European Medicines Agency. Available at: https://www.ema.europa.eu/en/documents/scientific-guideline/guideline-investigation-bioequivalence-rev1_en.pdf (Accessed July 5, 2023).

[B3] Davies M. J. Aroda V. R. Collins B. S. Gabbay R. A. Green J. Maruthur N. M. (2022). Management of hyperglycaemia in type 2 diabetes, 2022. A consensus report by the American diabetes association (ADA) and the European association for the study of diabetes (EASD). Diabetologia 65 (12), 1925–1966. 10.1007/s00125-022-05787-2 36151309 PMC9510507

[B4] Gregg E. W. Zhuo X. Cheng Y. J. Albright A. L. Narayan K. M. Thompson T. J. (2014). Trends in lifetime risk and years of life lost due to diabetes in the USA, 1985-2011: a modelling study. Diabetes & Endocrinol. 2 (11), 867–874. 10.1016/S2213-8587(14)70161-5 25128274

[B5] Guariguata L. Whiting D. R. Hambleton I. Beagley J. Linnenkamp U. Shaw J. E. (2014). Global estimates of diabetes prevalence for 2013 and projections for 2035. Diabetes Res. Clin. Pract. 103 (2), 137–149. 10.1016/j.diabres.2013.11.002 24630390

[B6] Hemmingsen B. Lund S. S. Gluud C. Vaag A. Almdal T. P. Hemmingsen C. (2013). Targeting intensive glycaemic control versus targeting conventional glycaemic control for type 2 diabetes mellitus. Cochrane database Syst. Rev. (11), CD008143. 10.1002/14651858.CD008143.pub3 24214280

[B7] Holman R. R. Paul S. K. Bethel M. A. Matthews D. R. Neil H. A. (2008). 10-year follow-up of intensive glucose control in type 2 diabetes. N. Engl. J. Med. 359 (15), 1577–1589. 10.1056/NEJMoa0806470 18784090

[B8] ICH (2016). Integrated addendum to ICH E6(R1): guideline for good clinical practice E6(R2). Available at: https://www.gmp-compliance.org/files/guidemgr/E6_R2__Step_4.pdf (Accessed July 5, 2023).

[B9] Korsatko S. Deller S. Koehler G. Mader J. K. Neubauer K. Adrian C. L. (2013). A comparison of the steady-state pharmacokinetic and pharmacodynamic profiles of 100 and 200 U/mL formulations of ultra-long-acting insulin degludec. Clin. drug Investig. 33 (7), 515–521. 10.1007/s40261-013-0096-7 23749405

[B10] Lipscombe L. L. (2014). The US diabetes epidemic: tip of the iceberg. Diabetes & Endocrinol. 2 (11), 854–855. 10.1016/S2213-8587(14)70172-X 25128273

[B11] Mobasseri M. Shirmohammadi M. Amiri T. Vahed N. Hosseini Fard H. Ghojazadeh M. (2020). Prevalence and incidence of type 1 diabetes in the world: a systematic review and meta-analysis. Health Promot. Perspect. 10 (2), 98–115. 10.34172/hpp.2020.18 32296622 PMC7146037

[B12] UK Prospective Diabetes Study (UKPDS) Group (1998). Intensive blood-glucose control with sulphonylureas or insulin compared with conventional treatment and risk of complications in patients with type 2 diabetes (UKPDS 33). UK Prospective Diabetes Study (UKPDS) Group. Lancet (London, Engl. 352 (9131), 837–853. 10.1016/S0140-6736(98)07019-6 9742976

[B13] US Food and Drug Administration (2009). Bioavailability and bioequivalence requirements. 21 CFR 21 Part 320. Available at: http://www.accessdata.fda.gov/scripts/cdrh/cfdocs/cfcfr/cfrsearch.cfm?cfrpart=320 (Accessed July 5, 2023).

[B14] US Food and Drug Administration (2015). Pharmacology review(s) for Tresiba. Available at: https://www.accessdata.fda.gov/drugsatfda_docs/nda/2015/203313Orig1s000_203314Orig1s000PharmR.pdf (Accessed July 5, 2023).

[B15] World Medical Association (2013). World Medical Association Declaration of Helsinki: ethical principles for medical research involving human subjects. JAMA 310 (20), 2191–2194. 10.1001/jama.2013.281053 24141714

